# Prof. Samuel G. Armour

**Published:** 1853-01

**Authors:** 


*Prof. Samuel G. Armour.* — Prof. Armour, late of the University of Iowa, and editor of the Western Medico-Chirurgical Journal, has removed to Cleveland, Ohio.

